# Mixed methods assessment of personal heat exposure, sleep, physical activity, and heat adaptation strategies among urban residents in the Boston area, MA

**DOI:** 10.1186/s12889-022-14692-7

**Published:** 2022-12-10

**Authors:** Chad W. Milando, Flannery Black-Ingersoll, Leila Heidari, Ibrahim López-Hernández, Julie de Lange, Abgel Negassa, Alina M. McIntyre, M. Pilar Botana Martinez, Roseann Bongiovanni, Jonathan I. Levy, Patrick L. Kinney, Madeleine K. Scammell, M. Patricia Fabian

**Affiliations:** 1 Department of Environmental Health, School of Public Health, Boston University, 715 Albany St, Boston, MA 02118 USA; 2 GreenRoots Inc, Chelsea, MA 02150 USA; 3 Institute for Global Sustainability, Boston University, Boston, 02118 USA

**Keywords:** Heat exposure, Mixed methods research, Community-academic partnership, Remote data collection

## Abstract

The growing frequency, intensity, and duration of extreme heat events necessitates interventions to reduce heat exposures. Local opportunities for heat adaptation may be optimally identified through collection of both quantitative exposure metrics and qualitative data on perceptions of heat. In this study, we used mixed methods to characterize heat exposure among urban residents in the area of Boston, Massachusetts, US, in summer 2020. Repeated interviews of *N* = 24 study participants ascertained heat vulnerability and adaptation strategies. Participants also used low-cost sensors to collect temperature, location, sleep, and physical activity data. We saw significant differences across temperature metrics: median personal temperature exposures were 3.9 °C higher than median ambient weather station temperatures. Existing air conditioning (AC) units did not adequately control indoor temperatures to desired thermostat levels: even with AC use, indoor maximum temperatures increased by 0.24 °C per °C of maximum outdoor temperature. Sleep duration was not associated with indoor or outdoor temperature. On warmer days, we observed a range of changes in time-at-home, expected given our small study size. Interview results further indicated opportunities for heat adaptation interventions including AC upgrades, hydration education campaigns, and amelioration of energy costs during high heat periods. Our mixed methods design informs heat adaptation interventions tailored to the challenges faced by residents in the study area. The strength of our community-academic partnership was a large part of the success of the mixed methods approach.

**Supplementary Information:**

The online version contains supplementary material available at 10.1186/s12889-022-14692-7.

## Introduction

The growing frequency, intensity, and duration of extreme heat events necessitates increased focus on reducing heat exposures [1]. Heat exposure is associated with numerous adverse health outcomes, including poor sleep quality, premature mortality, and health care utilization due to cardiovascular disease, renal disease, kidney stones, and diabetes [2]. These heat-related outcomes disproportionately occur among outdoor workers, older populations, individuals experiencing homelessness, Black adults and people with lower socio-economic status [3, 4]. Temperatures are elevated in urban neighborhoods due to higher retention of heat from the sun in dark and impervious surfaces, a phenomenon known as the urban heat island effect [5]. Furthermore, residents in these communities may lack adaptive infrastructure to reduce the risk of adverse impacts (e.g., air conditioners or access to community cooling centers).

Common techniques for assessing temperature exposures may not accurately characterize personal exposures amenable to intervention, especially among vulnerable populations. The majority of heat-and-health studies rely on meteorological data from outdoor weather stations, remotely sensed imagery, or statistical models [6], which each are proxies for exposure and typically do not include variation in individual vulnerabilities nor temperature extremes at a fine scale [7–9]. A long study duration is essential for establishing a counterfactual baseline to exposures during unplanned extreme heat events. Some recent studies have measured stationary temperature measurements as personal exposure proxies over long durations – for three months in homes in Detroit, Michigan, US [10], and for six weeks at traffic stops in Ahmedabad, Gujarat, India [11]. Characterizing risks related to heat using quantitative temperature metrics alone omits insight on psychological, physiological, behavioral, and social factors that modify the heat and health relationship for individuals [12]. Mixed research methods that integrate qualitative and quantitative data provide an avenue for holistically informing heat resiliency interventions across vulnerable individuals’ differing experiences of heat exposure and adaptation [13].

We used mixed research methods to assess individual experiences of heat exposure and adaptation during August and September of 2020 in two urban communities in the Boston area of Massachusetts. This work represents the first phase of the Chelsea and East Boston Heat Study (C-HEAT), a partnership between a local environmental justice organization and an academic institution. The goal of C-HEAT is to pursue strategies relevant to heat adaptation, coordinated with residents, city officials from various municipal branches, and non-profit organizations.

## Methods

### Project location

We conducted this study in the City of Chelsea (situated northeast of the City of Boston) and the East Boston neighborhood of the City of Boston (Figure S1), adjacent localities that rank third and fifth respectively as the most environmentally overburdened communities in Massachusetts [14]. Both communities experience urban heat island effects [15], and prior survey results indicated residents may be less aware of heat exposure risks and may lack the economic and political resources to cope with extreme heat [16, 17].

### Participant recruitment

Participant recruitment was led by GreenRoots, Inc., a grassroots environmental justice organization that has worked in Chelsea and East Boston for the last two decades [18], including in partnership with investigators at Boston University School of Public Health (BUSPH) [19, 20]. GreenRoots widely distributed digital and physical bilingual English and Spanish recruitment materials to GreenRoots members and in the communities. Inclusion criteria were being at least 18 years old, owning a smartphone and being willing to use it for research purposes, English or Spanish language proficiency, residing in Chelsea or East Boston at current residence for greater than one year, and planning to live at current residence for at least 6 months. We aimed to recruit up to 30 participants for this study. The Boston University Medical Campus Institutional Review Board approved all study protocols.

### Data collection and analysis

#### Qualitative interview data

**Fig. 1:**
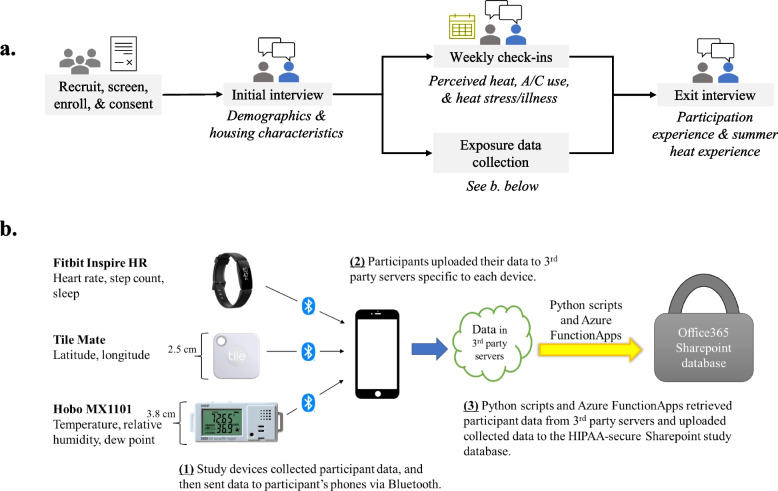
Schematic of (**a**) mixed methods study design and (**b**) remote exposure, location, and biometric data collection methods. Participants were given the three exposure collection devices described on the left side of panel (**b**)

We conducted an initial baseline interview, short weekly check-in interviews, and an exit interview (Fig. 1a). All were highly structured and included closed and open-ended questions, designed based on previous studies as well as topics of interest to community organizations [20–25]. All interviews were conducted via Zoom in English or Spanish, with participant responses entered into Qualtrics™. The baseline interview included questions about residential air conditioning, daily activities, sleep and hydration habits, occupation, transportation, health, and attitudes and behaviors for coping with the heat. The weekly check-in assessed thermal comfort and control [21, 22]. The exit interview asked about the experience of participating in the study. (See Supplemental Materials for interview guides).

#### Personal heat exposure, environmental temperature, and biometrics

We collected location, biometric, and temperature data for each participant using Bluetooth-enabled low-cost sensors with minimal battery requirements, and developed software to retrieve sensor data (Fig. 1b). Participant location was recorded every 10 min using a Tile Mate (San Mateo, California, US), with a horizontal position accuracy specific to each participant’s smartphone, e.g., 7 to 13 m for an iPhone 6 [26]. The Mate was attached to a keychain given to each participant. One-minute interval heart rate and step count, and nightly sleep data were collected via a Fitbit Inspire HR sport watch (Fitbit, San Francisco, CA, US). Recent evaluation of the predecessor to the Inspire HR showed a mean absolute error in heart rate of 7.3 bpm when at rest and 12.8 bpm during activity [27], and a slight underestimation of step counts, approximately 12.5% in a cohort of healthy older adults [28]. A meta-analysis showed that the Fitbit sleep data algorithm provides an adequate summary of nightly sleep patterns [29]. Temperature was recorded every 10 min using HOBO meteorological sensors (Onset, Cape Cod, MA, US) in the personal, indoor, and outdoor environments. For personal and indoor measurements, we used the HOBO MX1101, which has an accuracy of ± 0.2 °C (from 0° to 50 °C). We attached the personal HOBO to the keychain containing the Tile Mate. We remotely guided the participants through installing the indoor HOBO on a wall in the participant’s bedroom away from any air conditioning (AC) unit or window. We captured outdoor meteorological data by placing 30 outdoor HOBO sensors on streetlamps and street-adjacent trees in Chelsea and East Boston. We compared outdoor temperature and relative humidity measurements to concurrent data collected at a local National Weather Service (NWS) station at the Boston Logan International Airport (Latitude: 42.361° N; Longitude: -71.01° W). We developed Python scripts to retrieve participant data from third-party online databases and uploaded to HIPAA-compliant Office365 Sharepoint Lists. Ten-minute temperature measurements were averaged to create hourly estimates for analysis. Further details about the methods and data cleaning are available in the Supplemental Methods.

#### Analysis

For this analysis, we highlighted key findings related to heat resiliency interventions, using a range of temperature metrics. We examined differences in hourly personal, indoor, and outdoor temperatures during times when participants were within 5 km of their home address (Temperature metrics). We used the non-parametric Wald test to compare differences in hourly temperatures at various distribution percentiles [30], and the Spearman correlation coefficient to examine correlations in temperature metrics. We examined the impact of weekly (self-reported) AC use on indoor daily maximum temperatures using a linear mixed effect model, with fixed effects for outdoor maximum temperature, weekly AC use, an interaction term for outdoor maximum temperature and AC use, and a random intercept by participant (Air conditioning use and efficacy). We examined differences in sleep duration (measured by FitBit) when participants were at home (i.e., within 100 m of their home address) as a function of mean indoor temperature during each participants’ sleep hours (estimated from FitBit data), using linear mixed effects modeling and controlling for weekday and weekend (Variations in sleep with indoor temperature). We summarized heterogeneity in time away from home on hot days, defined as days with maximum daytime hourly outdoor temperatures above 26.5 °C (80 °F, a common temperature threshold for extreme heat [31]), and added context with information from baseline and weekly surveys (Variations in physical activity with outdoor temperature).

Finally, we analyzed all interview responses for key themes and prepared descriptive statistics to highlight opportunities for heat resiliency and adaptation strategies (Participant heat experiences and heat adaptation practices). All statistical analyses were conducted in R (version 4.1.3) [32]; linear mixed effects modeling was accomplished using the package lmer [33].

## Results

We enrolled and conducted baseline interviews with 24 participants, most of whom lived in Chelsea, were female, self-identified as Hispanic/Latina/o, and were renters in multi-family housing (Table 1). One participant was unable to complete the device configuration process, and another was lost to follow-up. Therefore, we collected temperature and biometric data with 22 of 24 participants for between 6–8 weeks. Data collected using our methodology demonstrated good quality and interpretable outputs, with data capture ranging from 75 to 96% of expected. Further details on participant recruitment and data cleaning are available in the Supplemental Results. In the following sections, we present findings that leverage our mixed methods approach and provide useful insights for future heat resiliency data collection and intervention efforts in the Boston Area.

**Table 1:** Participant demographics and housing characteristics (*N* = 24)

Variable	N	(%)
*Home city:*		
Chelsea	17	(71)
East Boston	7	(29)
*Sex:*		
Female	18	(75)
Male	6	(25)
*Race/Ethnicity:*		
Hispanic/Latina/o	11	(46)
Non-Hispanic White	6	(25)
Other ^a^	7	(29)
*Age (years):*		
22 – 30	5	(21)
31 – 39	7	(29)
40 – 64	8	(33)
65 – 78	4	(17)
*Language:*		
Spanish	11	(46)
English	13	(54)
*Foreign-born:*		
Yes	11	(46)
No	13	(54)
*Housing type:*		
Multi-family/Apartment	22	(92)
Single family	2	(8)
*Rent/Own*		
Rent	20	(83)
Own	4	(17)
*Air Conditioning type:*		
Window/Wall	17	(71)
Central	7	(29)

^a^includes American Indian or Alaska Native, Asian, Black or African American, and unknown

### Temperature metrics 

The median ambient hourly temperature was 20.6 °C, with peaks in the early afternoon of each day and lows in the early morning. On average, the distribution of personal hourly temperatures was largely higher than those measured outdoors in the study area and at the NWS station (Table 2), but similar to temperatures measured indoors (reflecting the high percentage of time spent in indoor residential environments). At the 50th percentile of hourly temperature distributions, personal temperatures were higher than outdoor local and NWS monitors by 2.9 (95% CI: 2.3, 2.5) and 3.9 °C (95% CI: 3.3, 4.5), respectively. The largest differences in temperatures occurred at the 5th and 25th percentiles, indicating that personal temperatures were elevated especially at the lower end of the distribution – likely reflecting differences at nighttime and the temperature lag between indoor and outdoor environments. Personal temperatures were not well correlated over time with outdoor temperatures, with Spearman correlation coefficients of 0.14 and 0.14 with outdoor local and NWS temperatures (for comparison, the Spearman correlation coefficient between outdoor local and NWS was 0.51, and between personal and indoor was 0.50). Similarly, indoor temperature was not well correlated with outdoor temperature (*R*^2^ = 0.13).

**Table 2:** Personal, indoor, outdoor, and nearest weather station temperature values and differences at the 5^th^ to 95^th^ percentile for hourly temperature distributions recorded over summer 2020

	Percentile				
	5^th^	25^th^	50^th^	75^th^	95^th^
Personal temperature (°C)	19.4	22.4	24.5	26.1	28.9
Indoor temperature (°C)	19.5	22.6	24.3	25.8	28.2
Outdoor local temperature (°C)	15.7	19.1	21.5	24.0	27.9
Outdoor NWS temperature (°C)	15.0	18.1	20.6	22.8	27.2
Difference (°C) between personal and indoor temperature (95% CI)^a^	-0.1 (-0.5, 0.2)	-0.2 (-0.6, 0.2)	0.2 (-0.04, 0.5)	**0.4 (0.1, 0.6)**	**0.8 (0.4, 1.1)**
Difference (°C) between personal and local temperature (95% CI)^a^	**3.7 (2.4, 4.9)**	**3.2 (2.5, 3.9)**	**2.9 (2.3, 3.5)**	**1.9 (1.4, 2.4)**	0.9 (-0.3, 2.1)
Difference (°C) between personal and NWS temperature (95% CI)^a^	**4.4 (3.1, 5.6)**	**4.3 (3.7, 5.0)**	**3.9 (3.3, 4.5)**	**3.3 (2.8, 3.8)**	**1.7 (0.5, 2.9)**

*NWS* national weather station, *CI* confidence interval

^a^Differences at various percentiles were calculated using the Wald Test (significant differences are bolded)

### Air conditioning use and efficacy

**Fig. 2:**
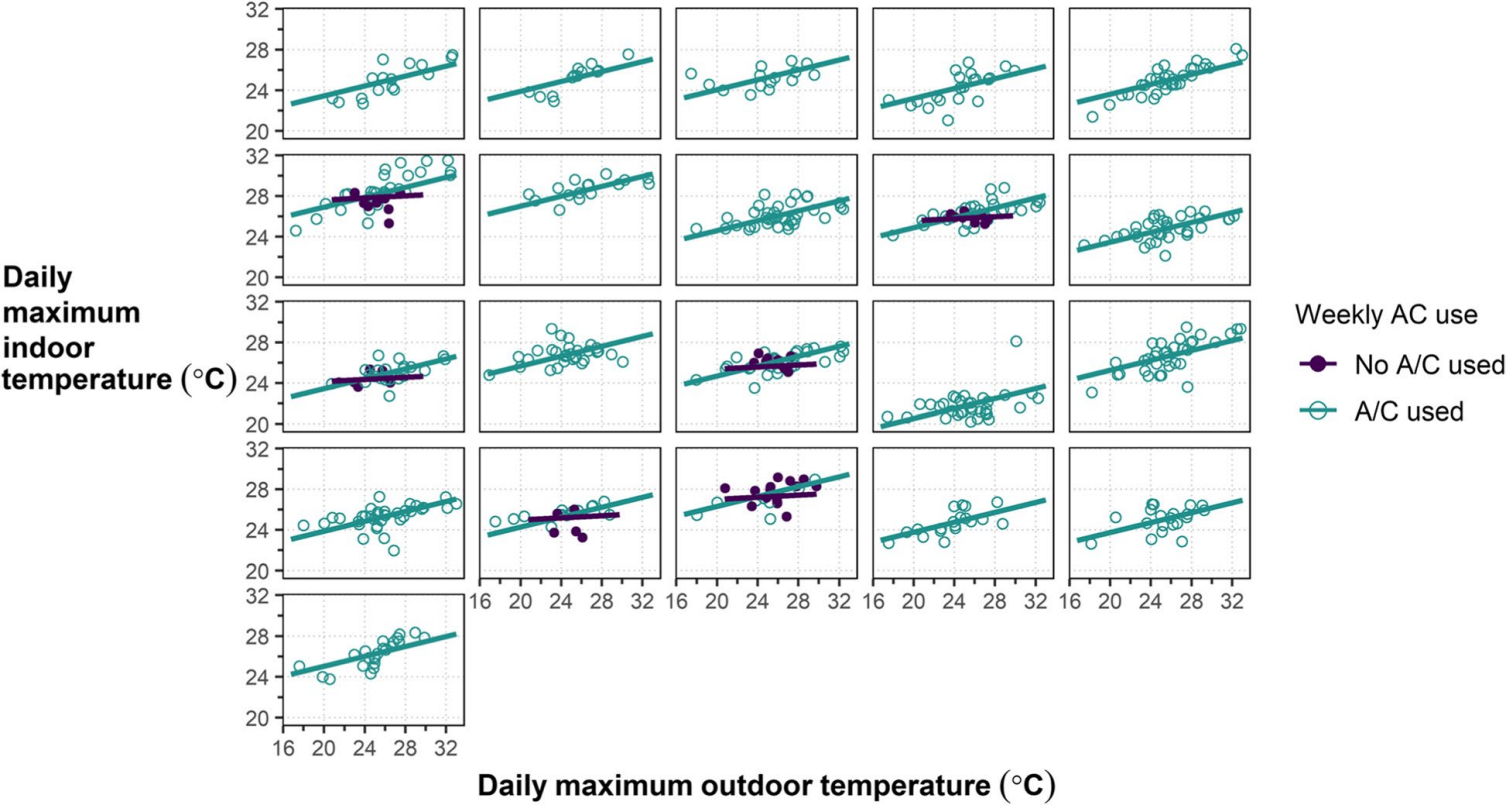
Participant level indoor versus outdoor maximum daily temperatures, stratified by AC used (*n* = 513 days) and no AC used (*n* = 50 days). Plot generated from linear mixed effects regressions with random intercept by participant

AC units for participants in this study did not adequately control indoor temperatures to desired thermostat levels. All participants reported having some form of AC (with 17 of 24 participants having window or wall units), however, 19 of 24 participants still described conditions in their homes at baseline as either “warm” or “hot.” Of participants with wall or window AC units, only 4 reported that their AC units were adequate to keep them cool enough. AC use was common throughout the study duration: only 6 participants reported not using their AC during a weekly interview, with 7 weeks total of no AC use across all participants. However, when AC was used (513 of 563 days), indoor maximum temperatures still increased with increasing outdoor temperatures (Fig. 2), quantified as a regression slope with a 0.24 °C increase in indoor maximum temperature per 1 °C increase in outdoor maximum temperature (95% CI: 0.22, 0.27). Most maximum daily indoor temperatures were well above the highest reported temperature AC setpoint from the baseline interview, 23.8 °C (75°F), and many reported setpoints were much lower (15.5 to 18.3 °C). No plateau of indoor temperatures was reached when AC units were in use; such a plateau would indicate that an AC unit had enough cooling capacity to maintain an indoor temperature regardless of increased outdoor temperature. For the limited times when AC was not used (50 of 563 days), there was relatively little change in indoor maximum temperature as a function of outdoor temperature (the regression slope was a 0.05 °C increase in indoor maximum temperature per 1 °C increase in outdoor maximum temperature, with 95% CI: -0.14, 0.24). This result is likely biased by temporality (which we were not powered to control for): all but two no-AC weeks were at the end of September when outdoor maximum temperatures occurred in narrower range than in mid-summer periods, and when nighttime temperatures were cool enough to alleviate the need for nighttime AC.

### Variations in sleep with indoor temperature

**Fig. 3:**
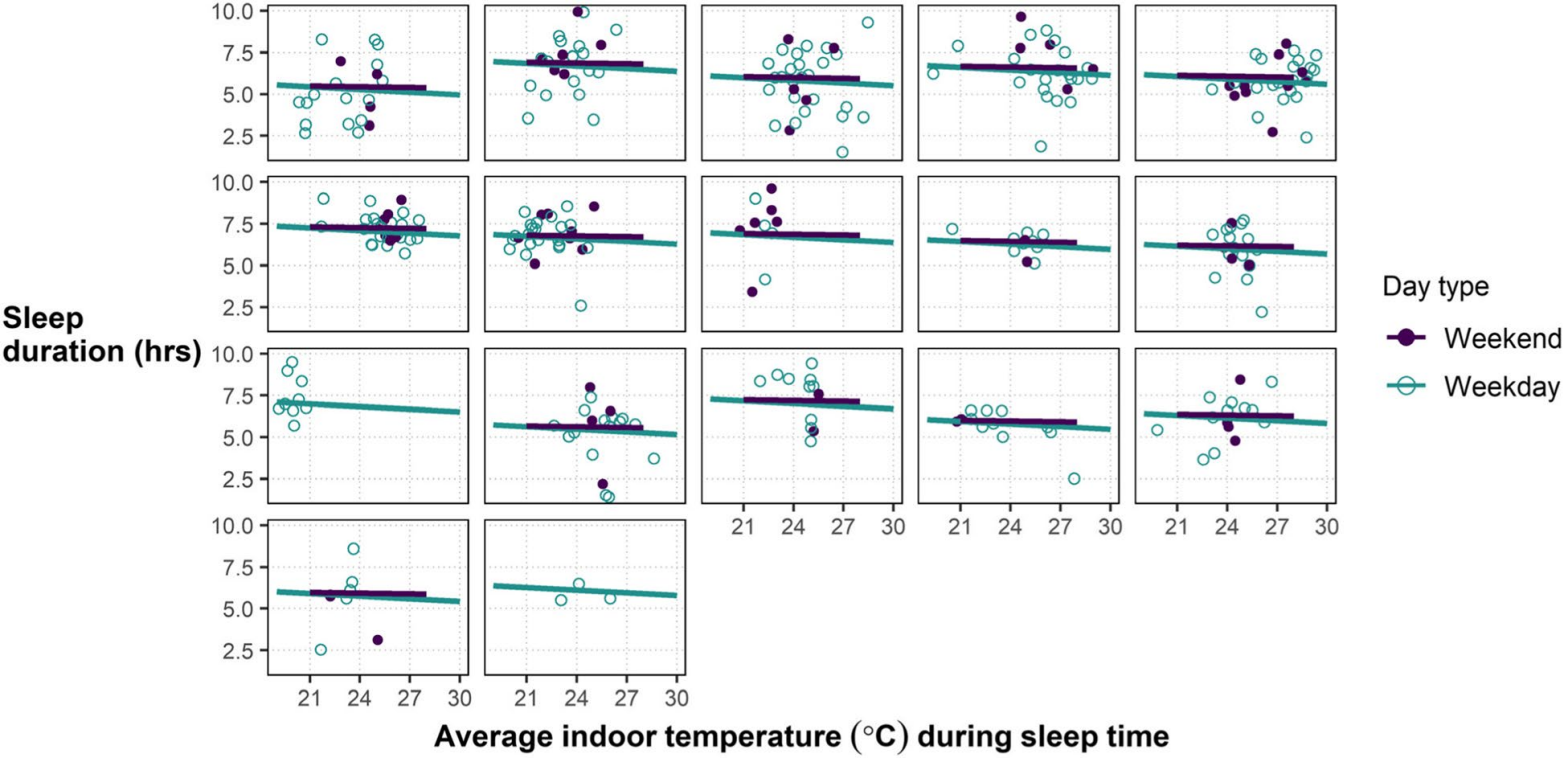
Sleep duration (hrs) and average bedroom temperature during sleep time (°C) of *N* = 308 person-nights where participants were within 100 m of their home address

We did not observe a statistically significant relationship between higher indoor temperatures and reduced sleep duration (Fig. 3). For example, on weeknights (n = 235 nights, an average of 13.8 nights per person), the fixed effect of regression slope was -0.05 h of sleep per 1 °C increase in mean indoor temperature during sleep time (95% CI: -0.16, 0.05). We did not control for many individual determinants of sleep, including time of sleep onset (e.g., 11 pm versus 2am), which undoubtedly influenced model performance. The sleep findings from weekly questionnaires offered a similar picture to these quantitative findings, as most participants did not report major disruptions in weekly sleep. Out of 148 weekly check-ins (average of ~ 6 per participant) participants reported ‘more disrupted than usual’ sleep 26 times, ‘better than usual’ sleep 25 times, and ‘same as usual’ sleep 95 times. However, a total of 13 participants reported disrupted sleep at least once, indicating some heterogenity in sleep quality across participants. Visual inspection of weekend and weekday plots indicates clustering and non-linearity in sleep times per person as a function of indoor temperature.

### Variations in physical activity with outdoor temperature

**Fig. 4:**
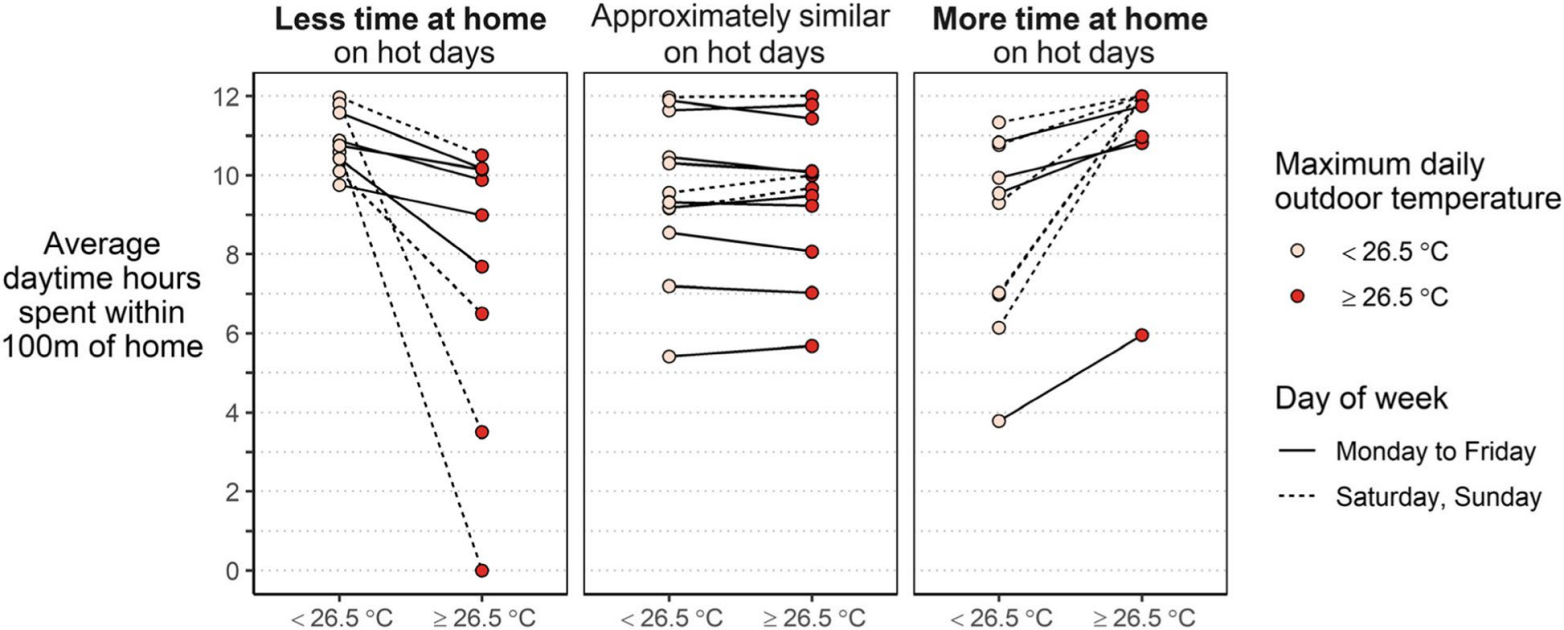
Difference in a participant’s average time spent within 100 m of home on days when the maximum daily temperature was above or below 26.5 °C. Each pair of connected points represents data for a single participant. If the average difference in time spent at home was less than 30 min, the connected points were labeled ‘Approximately similar’. If the difference in time spent at home exceeded 30 min, the connected points were grouped into the ‘Less time at home’ or ‘More time at home’ categories, respectively. Participants may have lines in multiple panels, as data are grouped by weekday vs. weekend

Trends between temperature and daytime physical activity were difficult to discern, although there were some visually observable patterns (Fig. 4). On weekdays where the daily maximum temperature was greater than 26.5 °C, 5 of 18 participants spent less time at home (by an average of 30 min or more), 4 of 18 participants spent more time at home (by an average of 30 min or more), and 9 participants stayed at home for approximately the same amount of time on average (average difference within 30 min). We observed similar groupings on weekends, although there were fewer weekend days with data and fewer weekend days with maximum temperatures above 26.5 °C. These strata are descriptive and reflect the cutpoints used (26.5 °C, 30 min differences, within 100 m of home); still, they describe a range of behavior patterns related to heat that could be considered in future planning. Qualitative data support this finding of heterogeneous behavior changes on hot days; 11 of 24 participants reported intentionally changing modes of transportation on hotter days. On 19 of 148 weekly total check-ins, a participant included ‘Leave the house for a cooler area’ as one of their first three strategies used to cool down on a hot day. Participants who did leave their homes to cool off reported seeking either a friend’s home or business with AC, or going outside for a walk or to a park.

### Participant heat experiences and heat adaptation practices 

Qualitative data revealed several additional opportunities for heat adaptation interventions. Concerns related to heat were varied; 10 participants reported “not being concerned at all” about their own personal health risks related to heat, nine participants reported either being “moderately” or “extremely” concerned about personal heat-related illness, and 16 of 24 participants reported feeling worried for others in their household or community. Nine participants reported having to make choices about which bills to pay, and nine also reported that extreme temperatures influence which bills are paid (with some overlap in the two responses). The most popular heat adaptation strategies were using AC, removing clothing, and opening windows. While using window shades can decrease indoor solar heat gain, it was ranked as one of the least popular strategies, suggesting the potential for cooling interventions that include installation of these devices in homes. Roughly half of our participants (13 of 24) reported not ever leaving their homes to seek a cool shelter, indicating that for some, the home is the primary place of refuge during extreme heat events. Adequate hydration is an essential heat adaptation strategy, however, 10 of 24 participants reported not drinking enough water, based on their own determination of “enough”. During exit interviews, participants reflected on increased awareness of heat-related illness, identification of barriers to keeping cool (e.g., utility cost, difficulty staying hydrated, difficulty finding cool places to go outside the home), and provided valuable feedback for intervention suggestions (e.g., subsidies for utility costs on hot days, desire for greater access to and availability of local parks).

## Discussion

We used mixed research methods to integrate numerous data sources on heat exposure, heat adaptation strategies, and sleep and physical activity variation from late summer to early fall 2020 in the Boston area. The variability in our findings supports the utility of capturing personal-level data, as personal-level temperatures were the highest recorded of any data stream and were not correlated in time with ambient metrics. AC units did not adequately control indoor temperatures. Sleep duration was not associated with warmer indoor temperatures. On warmer days, we observed a range of percent time-at-home, expected given our small study size. Exit interviews identified several possible intervention topics, including hydration, window shades, correct sizing of AC, subsidized energy bills for AC use, and facilitating cooler modes of transportation. Qualitative data streams contextualized analyses and provided insights into adaptive behaviors not captured in the quantitative data. These insights are essential for tailoring heat resiliency interventions for specific populations and locations.

The mixed methods approach of the study was strengthened by the community-academic partnership, which allowed us to navigate challenges of recruitment and retention in predominantly low-income neighborhoods during the onset of the COVID-19 pandemic. The COVID-19 prevalence in Chelsea and East Boston was many times higher than neighboring communities [34]. Income, immigration status, and education in majority low-income and Hispanic communities, the “most digitally underserved in the U.S.” [35], modify a family’s ability to adopt new technologies [36]. These factors necessitated developing strong relationships with study participants responsible for setting up and collecting their personal data. In addition, this partnership enhanced the feasibility of remote qualitative and quantitative data collection methods, which are generalizable to other studies in resource-limited or physically distant settings and can serve to contextualize public health interventions with participant experiences.

### Limitations

Our protocols yielded substantive high-quality data with some limitations in the methods and impacts on generalizability. Incorporating more open-ended interview questions would have better captured the nuances of participants’ experiences and current adaptation practices for extreme heat. Requiring ownership and operating proficiency of a smartphone biases the population sample towards individuals more likely to be able to accomplish the study methods. Smartphone settings and device permissions sometimes changed during the study due to automatic app updates, limiting data capture temporarily. Biometric and location data were at times challenging to collect given a range of device failures and the challenges of maintaining participant compliance with remote study protocols. The small number of participants varied in age, occupation, baseline physical activity level, housing, and daily time-activity patterns, which made it challenging to identify participant-wide patterns in complex biometric data related to heat. Despite these limitations, real-time data collection and quality control methods allowed us to minimize data gaps and remotely capture temperature measurements and biometric data. Also, our heterogeneous participants provided a wide range of qualitative answers useful for intervention planning. Where possible, future studies on heat adaptation should capture quantitative data on adaptation use characteristics, allow for opened ended survey responses to add further context, conduct a pre-study feasibility assessment of sensors, and implement a sensor usage schedule to support participants with varying levels of technological comfort [37].

Our findings were also limited by external circumstances. The physical distancing mandates in the early summer 2020 phase of the COVID-19 pandemic forced us to transition all methods to remote, postponing the study timeline from our initial start date of early June. We were unable to recruit up to 30 participants due to our limited capacity to effectively engage participants virtually during the pandemic. It took more time with each participant than would have been the case if we could go to their homes and install the sensors. This may also have influenced participant engagement, with meetings and interviews held online, then still a new format for participants and researchers alike. That said, our generally robust data capture and interpretable findings reinforce the viability of remote qualitative and quantitative data capture for heat and other exposures.

### Comparison to the literature

Our study contributes to the growing body of research using mixed methods to study heat-related exposures, resiliency, health awareness, and health outcomes. A mixed methods study of extreme heat vulnerabilities among older adults in Canada reported the importance of access to resources mitigating heat, although heat exposures were not quantified among study participants [38]. Awareness of the health risks of high heat was assessed among a cohort of residents in Knoxville, TN, with only 55% of respondents reporting concerns about the health risks of extreme heat [39]. Our specific finding about AC usage and efficacy has been demonstrated in other work. AC use as a mitigating strategy was reported qualitatively and quantitatively among older study participants in a cohort in Australia; however, outdoor temperatures were much higher than in this study (exceeding 38 °C), and AC units were successful in achieving an indoor plateau of temperatures [40]. Similarly, a Baltimore, MD study demonstrated AC efficacy among low-income residences with central air conditioning [41]. These studies, and ours, indicate opportunities to tailor local adaptation to AC use, develop public cooling services, and educate on the health impacts of extreme heat. Subsequent in-depth interviews with a subset of C-HEAT Study participants provided further insight into the financial decision-making regarding AC usage, reasons for inadequate hydration at the workplace and at home (lack of sanitary facilities, fear of job loss, and low confidence in water safety), and heat related health concerns and behaviors [42].

Our sleep findings differ from the literature, likely due to the heterogeneity of our participant population and modest sample size to evaluate the association Other studies of sleep and heat have focused primarily on older adults. A study of sleep quality among elderly participants in Shanghai, China, found that increased air temperature was correlated with decreased sleep efficiency and time asleep [43]. In a Northeastern US study of indoor temperatures and health in a cohort of older adults, researchers reported more disrupted sleep and increased heart rates at higher indoor temperatures [44].

Studies of personal heat exposure also use a range of study designs and temperature exposure metrics, which limits comparability between results. Some studies that characterize exposures using personal temperature measurements collect data for a week or shorter, or in occupational settings, which differ greatly from residential settings not only in temperature extremes but also in participant’s ability to control their surroundings [7, 8, 45–47]. Research examining correlations between personal, indoor, and ambient temperature metrics found a wide range of correlation coefficients (R^2^ from 0.21 to 0.39 for personal-ambient, 0.4 for indoor-ambient, highlighting the importance of estimating exposure in micro-environments and the importance of seasonality in correlation strength [48, 49]. In this study the correlation between personal and ambient temperature was lower (R^2^ = 0.14), as was the correlation between indoor and ambient temperatures (R^2^ = 0.13). A recent study found that time spent outside and income were more closely correlated with personal heat exposure than regional weather station observations [50]. This same study suggested standardizing temperature exposure metrics in order to better characterize exposure as well as compare exposure measurements across studies.

## Conclusions

Mixed research methods provide a mechanism to capture rich contextualized data on heat exposures and adaptation practices, even in studies with small sample sizes. Personal-level behavior, residential characteristics, and daily activity patterns dictate exposures and influence resultant health outcomes and may not be adequately estimated using ambient monitoring or survey data alone. Furthermore, understanding the current patterns of use and efficacy of current adaptation practices, e.g., AC units, provides opportunity to build and improve on existing interventions, both from a quantitative perspective (e.g., a more effective unit), and qualitative (e.g., providing a subsidy to offset high electricity costs during hot days, as identified by survey results). It is often the communities most vulnerable to heat exposures for whom data collection is most challenging; and thus, for which tailored resiliency interventions, characterized using mixed methods, can be most beneficial.

## Supplementary Information

**Additional file 1. **

## Data Availability

The datasets supporting the conclusions of this article are not publicly available in an online repository, but can be made available upon request to the lead author (Dr. Chad Milando, cmilando@bu.edu).
