# Supplementary material for: Mixed methods assessment of personal heat exposure, sleep, physical activity, and heat adaptation strategies among urban residents in the Boston area, MA
Source: BMC Public Health. 2022 Dec 10;22:2314. doi: 10.1186/s12889-022-14692-7 (PMC9739346; doi:10.1186/s12889-022-14692-7)
Supplement: Supplementary file 1 — Additional file 1. [file 12889_2022_14692_MOESM1_ESM.docx]

# Supplemental Materials

**Mixed methods assessment of personal heat exposure, sleep, physical activity and heat adaptation strategies among urban residents in the Boston area, MA.**

Chad W. Milando ^a^, Flannery Black-Ingersoll ^a^, Leila Heidari ^a^, Ibrahim López-Hernández ^b^, Julie de Lange ^a^, Abgel Negassa ^a^, Alina M. McIntyre ^a^, M. Pilar Botana Martinez ^a^, Roseann Bongiovanni ^b^, Jonathan I. Levy ^a^, Patrick L. Kinney ^a^, Madeleine K. Scammell ^a^, M. Patricia Fabian ^a^

^a^ Department of Environmental Health, Boston University School of Public Health, 715 Albany St, Boston, MA, 02118, USA

^b^ GreenRoots Inc, Chelsea, MA, 02150, USA.

Supplemental includes:

- Supplemental Methods
- Supplemental Results
- Supplemental Tables
- Supplemental Figures
- Initial interview
- Weekly interview
- Exit interview

## Supplemental Methods

### Exposure data collection

Participant location was recorded every 10 minutes using a Tile Mate (San Mateo, California, US). The Mate communicates with a wireless device with Global Positioning System (GPS) or WiFi capability (e.g., a smartphone) to capture the wireless device’s last known location and uses the Tile smartphone app and the wireless device’s internet capabilities to transmit this location to the online Tile database. Smartphone location data vary in horizontal position accuracy, e.g., 7 to 13m for an iPhone 6^[[1]](#footnote-1)^. The Mate does not require calibration and has a one-year battery life. Participants attached the Mate to a keychain always kept on their person. We developed a Python script to retrieve participant location data from the Tile online database and upload location data to a HIPAA-compliant Office365 Sharepoint List. We ran this script every 10 minutes using a Microsoft Azure FunctionApp to create a database of participant locations. Throughout the study, we ran processing scripts to check collected data. Following identification of data gaps, we asked participants to reset location sharing privileges for the Tile app. At the end of the study, we downloaded the Tile Sharepoint List to a secure offline storage drive.

One-minute interval heart rate and step count, and nightly sleep data were collected via a Fitbit Inspire HR sport watch (Fitbit, San Francisco, CA, US). The Inspire HR has a five-day battery life and can store up to one week of biometric data between device syncs. Recent evaluation of the predecessor to the Inspire HR showed a mean absolute error in heart rate of 7.3 bpm when at rest and 12.8 bpm during activity ^[[2]](#footnote-2)^, and a slight underestimation of step counts, approximately 12.5% in a cohort of healthy older adults ^[[3]](#footnote-3)^. A meta-analysis showed that the Fitbit sleep data algorithm provides an adequate summary of nightly sleep patterns ^[[4]](#footnote-4)^. Participants were asked to wear the Inspire HR on their non-dominant wrist at all times, except when charging or showering, and uploaded their data during weekly check-ins. To retrieve these data, we developed a FitBit application, developed Python scripts to access participant-specific data via this application, and used FunctionApps to run these scripts and upload data to Sharepoint. We retrieved minutely heart rate and step count data every hour and retrieved sleep data once every other day and developed quality control scripts. We employed an Azure storage queue to enable asynchronous processing of incoming minutely heart rate and step count data. After the study concluded, we download minutely average participant data for the duration of the study.

Temperature was recorded every 10 minutes using HOBO meteorological sensors (Onset, Cape Cod, MA, US) in the personal, indoor, and outdoor environments. For personal and indoor measurements, we used the HOBO MX1101, which has an accuracy of ±0.2 °C (from 0° to 50°C). The on-device storage limit of the MX1101 is 84,650 measurements, and, with a logging interval of 10 minutes, device battery life is greater than one year. We attached the personal HOBO to the same on-person keychain as the Tile Mate. We remotely guided the participants through installing the indoor HOBO on a wall in the participant’s bedroom away from any AC unit or window. During weekly check-ins, we asked participants to open the HOBO mobile application and upload data to the HOBO web server. We checked participant-specific HOBO data dashboards twice a week to ensure data capture. We captured outdoor meteorological data by placing 30 outdoor HOBO sensors on streetlamps and street-adjacent trees in Chelsea and East Boston. Device models were MX2301, MX2302A, MX2304; all had similar accuracy, battery life, and on-device storage as MX1101, however model MX2034 only captured temperature. Land-use regression parameters and locations of interest identified by the community advisory team informed device placement. We considered device shading in the orientation of each sensor at each site. Sensors were installed three to four meters off the ground and were minimally secured against theft and vandalism via study logo stickers and contact information placed on the devices. Following the conclusion of the study, we downloaded data for each HOBO device to a secure offline storage drive.

### Data cleaning

We excluded Tile data points collected when location-sharing privileges on participant’s phones were reset and visually screened participant data for large discontinuities or points outside of New England. Tile data, recording latitude and longitude in 10-minute increments, were cleaned, with invalid and missing data identified. Data analyses were conducted for weekdays during the daytime (8:00:00 AM to 7:00:00 PM). After converting latitudinal and longitudinal coordinates from degrees to meters, based on the conversion units for Chelsea’s City Hall (Latitude: 42.39343° N; Longitude: -71.03346° W), we calculated the Euclidean distance from the participant’s home. Coordinates were flagged as ‘away from home’ when the distance was greater than or equal to 100 meters from the participant’s home. We then summed the number of 10-minute periods per day within 100 meters of home and converted the sum to hours.

For biometric data, we excluded any steps data without commensurate heart rate data. The Fitbit steps data were analyzed using methods similar to the Tile data. To discern whether there were differences in daily step count when comparing hotter days with cooler days, we summarized Fitbit steps to daily sum step counts, and compared these sums with the peak outdoor temperature of that day. Similar to other data streams, given the limited set of participants (and observations) for hotter and cooler days, respectively, we did not conduct statistical testing to discern whether there are significant differences in physical activity by temperature category.

For temperature data, we removed erroneous outliers in collected data (e.g., when the recorded temperature was 537.33 or -57.5 degrees Celsius). Once outliers were excluded, 10-minute temperature measurements were averaged to create hourly estimates for analysis.

## Supplemental Results

### Participant drop-out

We enrolled 24 of 30 total respondents to our survey. Two respondents were ineligible based on location and length of residence time, two respondents were eligible but contacted us late in the study process, and two were unable to enroll based on technological challenges.

### HOBO

We collected 121,020 personal HOBO temperature measurements (94% of expected), and 125,960 indoor HOBO measurements (96% of expected). Of 22 participants with personal and indoor sensors, three participants needed an additional session with the study team to finalize personal HOBO setup, and two participants needed an additional session to finalize indoor HOBO setup. Invalid data points were infrequent in both settings: we removed 159 observations in five of the personal HOBO 22 devices and 26 observations in three of the indoor HOBO 22 devices. Only five personal HOBO devices had an average percent capture below 99%; missing data occurred when the batteries for these devices fell out. Only three indoor HOBO devices had an average percent capture below 99%; missing data for these devices occurred via battery and configuration failures.

At the neighborhood level, we collected 177,236 outdoor HOBO measurements from 30 outdoor monitors (93% of expected). No outdoor HOBO data points were removed during data cleaning. Missing data occurred because four of the 32 outdoor sensors were lost during the sampling campaign, thus they only have partial data records.

### Fitbit

We collected 998,351 records of heart rate and step counts (75% of expected), and 727 person-nights of sleep data (75% of expected). For Fitbit data, no data points were removed during data cleaning. Only two participants had less than 50% average capture of expected daily heart rate and step count data, and only three had less than 50% capture of sleep data. Data gaps occurred if a participant did not wear their Fitbit or wore it too loosely. Two participants needed a second meeting with the study team to finalize Fitbit setup. Missingness patterns were not correlated with specific hours or days and were not correlated with missingness in location data.

### TILE

We collected 76,449 location data points, 85% of the total expected, from 18 of the 22 participants with exposure sensors (4 of the 22 participants with exposure sensors were not able to collect location data due to technological difficulties). Two of the 22 participants with exposure sensors not able to get the Tile app to function, leaving 20 participants with location data. With these 20 participants, we expected to collect 90,281 data points given the start date for each participant, the study end date, and the sampling frequency. We gathered 88,840 location data points in total and removed 12,391 that did not meet data quality checks (indicating that location data permissions needed to be reset). These invalid data points occurred for the entire record of two participants and for the beginning of the record for two others, leaving 18 participants with location data. Only two participants of these 18 had data capture less than 98%. Missing observations occurred not at random, due to Graph API overloading (i.e., too many requests in a given time period) or delays in the Tile API response. For all participants, location data from September 12 to 21 were lost due to a database saving error; these dates were excluded from the calculation of total expected data capture.

## Supplemental Figures


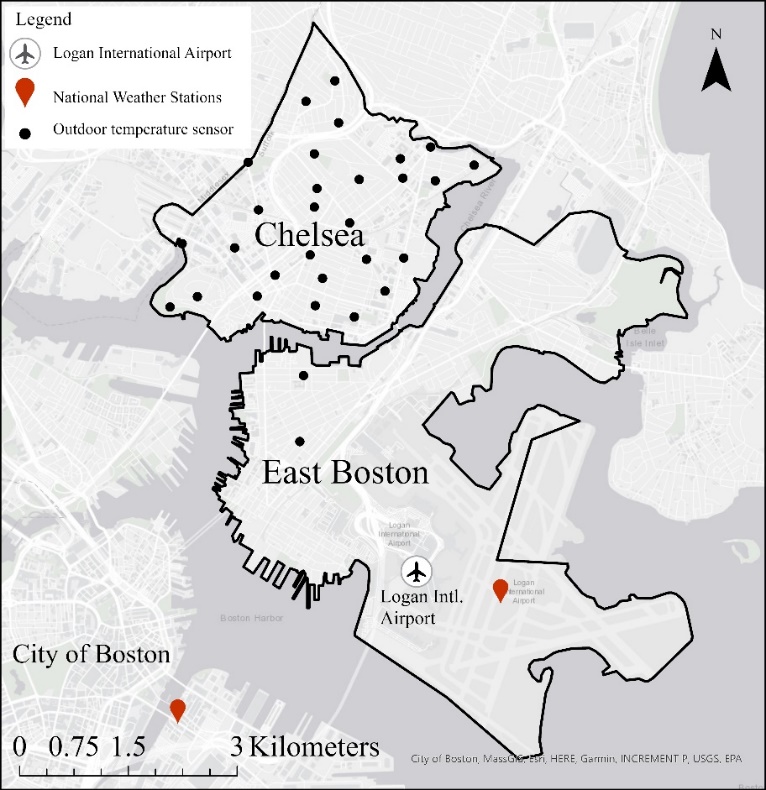


**Supplemental Figure 1**. Study area and outdoor temperature sensor placement.

## Initial interview

### Residence questions

We would like to start by asking you questions about your residence.

#### General

**1.** How long have you lived in this residence?

1. Less than 1 year
2. £ 1 to 2 years
3. £ 3 to 5 years
4. £ over 5 years

**2.** Do you rent or own this residence?

1. £ Own

2. £ Rent

3. £ Other arrangement, specify: 3a. ________________________________________

Examples may include: group home, staying with friends or family without paying rent, etc.

99. £ Don’t Know

88. £ Refused to answer

**IF YOU RENT**,

Are you… [READ EACH]

1. □ A public housing resident living in a building owned by the Housing Authority?
2. £ A resident of a building managed by a program or organization that provides affordable housing

2a. If so, which program? ____________________

1. £ Part of a household that receives rental assistance, such as a “Section 8” voucher
2. £ None of the above

99. □ Don’t Know

1. □ Refused to Answer

**3**. Including yourself, how many adults 18 years of age or older live in this residence? Count anyone who spent at least 3 nights per week here for the last month or longer:

1. Number of adults______________
2. Are any 65+ [ Y / N] if so how many ______

99. □ Don’t Know

88. Refuse to answer

**4.** How many children 17 years of age or younger live in this residence? Count anyone who spent at least 3 nights per week here for the last month or longer:

1. □ Number of children______________

99. □ Don’t Know

88. Refuse to answer

**5.** How would you describe typical living conditions in your residence during last SUMMER?

1. £ Hot
2. £ Warm
3. £ About right
4. £ Cool
5. £ Cold

99. □ Don’t know

88. Refuse to answer

#### Air conditioning

These next questions will ask about air conditioning in your residence.

**6.** Do you have functioning air conditioning anywhere in your home? [Select all that apply]

1. No.
2. Yes, Window AC (Skip to #7)
3. Yes, Wall AC (Skip to #7)
4. Yes, Portable AC (Skip to #7)
5. Yes, Central AC.
6. *Don’t know.*
7. *Refused*

**>> IF NO, “don’t have functioning AC”**

Why don’t you have air conditioning? Is it because… [Select all that apply]

1. You don’t need it
2. It’s too expensive
3. You want to conserve electricity
4. Your building’s wiring is not equipped to run an AC
5. You don’t like air conditioning
6. Building owner does not allow
7. Don’t know
8. Refused
9. Other: _______________________________

>> **skip to 1.3 Time activity**

**>> IF YES, have Central AC, continue here**

Do you use the central air conditioning?

1. □ Yes
2. £ No

88. Refuse to answer

**…………… >>> IF YES to “use CENTRAL AC”**

Ok, I will read several responses and ask for you to rate your agreement with the statement that, *your Central AC keeps you cool enough when it is on.* Would you say you…

**1**. Strongly disagree

**2**. Disagree

**3**. Somewhat agree

**4**. Agree

**5**. Strongly agree

[Don’t read]

**6**. Don’t know

**7.** Refused

During a typical week last summer, how often did someone in the household use central air conditioning?

1. Always
2. Most days of the week
3. About half the days of the week
4. Less than half the days of the week
5. Never
6. Don’t know
7. Refused

How many hours per day was it turned on (on average)?

a. Number of hours in the morning (8AM-12PM) __________ [min=0; max=4] £ Don’t Know

b. Number of hours in the afternoon (12 PM- 4 PM) _________ [min=0; max=4] £ Don’t Know

c. Number of hours in the evening (4 PM-10PM) __________ [min=0; max=6] £ Don’t Know

d. Number of hours at night (10 PM-8AM) __________ [min=0; max=10] £ Don’t Know

Recall the hottest / most uncomfortably hot day last summer. On this day, how many hours was your air conditioner (or central AC system) turned on?

a. Number of hours in the morning (8AM-12PM) __________ [min=0; max=4] £ Don’t Know

b. Number of hours in the afternoon (12 PM- 4 PM) _________ [min=0; max=4] £ Don’t Know

c. Number of hours in the evening (4 PM-10PM) __________ [min=0; max=6] £ Don’t Know

d. Number of hours at night (10 PM-8AM) __________ [min=0; max=10] £ Don’t Know

Do you typically set a temperature on your Central AC during the summer?

1. Yes
2. No
3. Don’t know
4. 88. Refuse to answer

If yes, at what temperature do you usually set the thermostat during the daytime (7am-7pm)?

[_____°F][_____ °C] or 99. £ Don’t Know 88. Refuse to answer

At what temperature do you usually set the thermostat during nighttime (7pm-7am)?

   [_____°F][_____ °C] or 99. £ Don’t Know 88. Refuse to answer

***>> Skip to section 1.3 Time Activity***

**…………… >>> IF NO to “use CENTRAL AC”**

You don’t use your central AC, why not? [Select all that apply]

1. Don’t need it / don’t feel hot.
2. You don’t like air conditioning
3. Prefer a fan.
4. Feel concerned that the electric bill would be too high.
5. Go somewhere else to get cool.
6. Feel like it would make a health condition worse (volunteer)
7. Don’t know
8. Refused
9. Other ______________________________________

**>> IF YES, have wall, window, or portable AC, continue here**

**7.** Do you use your window, wall or portable air conditioning units?

1. □ Yes
2. £ No

88. Refuse to answer

**…………… >>> IF YES to “use window wall or portable AC”**

Please describe the location and temperatures that you use with your units

| **AC type** | **In what rooms** | **Do you set the temperature?** | **What do you set the temperature to** |
| --- | --- | --- | --- |
| Wall |  |  |  |
| Window |  |  |  |
| Portable  *** do you carry the portable from room to room? [Y / N] |  |  |  |

Could you rate your agreement with this statement: Your window, wall or portable AC unit(s) keep(s) you cool enough when on:

**1**. Strongly disagree

**2**. Disagree

**3**. Somewhat agree

**4**. Agree

**5**. Strongly agree

***6****. Don’t know*

***7.*** *Refused*

Last summer how often did anyone in the household use air conditioning?

1. Always
2. Most of days of the week
3. About half the days of the week
4. Less than half the days of the week
5. Never
6. Don’t know
7. Refused

How many hours per day was it turned on (on average)?

a. Number of hours in the morning (8AM-12PM) __________ [min=0; max=4] £ Don’t Know

b. Number of hours in the afternoon (12 PM- 4 PM) _________ [min=0; max=4] £ Don’t Know

c. Number of hours in the evening (4 PM-10PM) __________ [min=0; max=6] £ Don’t Know

d. Number of hours at night (10 PM-8AM) __________ [min=0; max=10] £ Don’t Know

Recall the hottest / most uncomfortably hot day last summer. On this day, how many hours was your air conditioner (or central AC system) turned on?

a. Number of hours in the morning (8AM-12PM) __________ [min=0; max=4] £ Don’t Know

b. Number of hours in the afternoon (12 PM- 4 PM) _________ [min=0; max=4] £ Don’t Know

c. Number of hours in the evening (4 PM-10PM) __________ [min=0; max=6] £ Don’t Know

d. Number of hours at night (10 PM-8AM) __________ [min=0; max=10] £ Don’t Know

**…………… >>> IF NO to “use window wall or portable AC”**

If no, why not? [Select all that apply]

1. Don’t need it / don’t feel hot.
2. You don’t like air conditioning
3. Prefer a fan.
4. Feel concerned that the electric bill would be too high.
5. Go somewhere else to get cool.
6. Feel like it would make a health condition worse (volunteer)
7. Don’t know
8. Refused
9. Other ______________________________________

#### Time-activity

The next section will ask you (person being interviewed only) questions about the time you spend in and outside of your residence. We have divided the day into four time periods (morning, afternoon, evening and night). I’ll ask you about each, and tell you the hours included in each time period.

**8.** For a typical week *this* summer: [go through each day, each segment, going down for weekdays, and then repeat for Sat-Sun]

|  | **Monday - Friday** | | |  | **Saturday and Sunday** | | |
| --- | --- | --- | --- | --- | --- | --- | --- |
| **Please write in the number of hours spent (or check “Don’t Know):** | In your residence | Outside residence | Don’t know |  | In your residence | Outside residence | Don’t know |
| in the morning (8AM-12PM)  (Row must sum to 4 hrs total for Monday-Friday and for Saturday/Sunday) |  |  |  |  |  |  |  |
| in the afternoon (12 PM- 4 PM)  (Row must sum to 4 hrs total for Monday-Friday and for Saturday/Sunday) |  |  |  |  |  |  |  |
| in the evening (4 PM-10PM)  (Row must sum to 6 hrs total for Monday-Friday and for Saturday/Sunday) |  |  |  |  |  |  |  |
| at night (10 PM-8AM)  (Row must sum to 10 hrs total for Monday-Friday and for Saturday/Sunday) |  |  |  |  |  |  |  |

**8a.** Is this very different from last summer? [ yes / no / uncertain ]

**If yes,** is it because of COVID-19? [ yes / no / uncertain ]

What would your week and weekend days normally look like?

[we do not need details for every section of the day, but more to see how representative or wildly different this summer is compared to last.]

### Participant health questions

We would like to ask you questions about your (person being interviewed only) health. As a reminder, you do not have to answer any question you feel uncomfortable answering. I will start by asking about your overall health.

#### General

**9.** In general, would you say your health is: …

1. £ Excellent
2. £ Very good
3. £ Good
4. £ Fair
5. £ Poor

88. £ Refused

**10.** Has any health professional ever told you that you had any of the following chronic conditions? Please indicate Yes or No for each.

| **Condition** | **Yes** | **No** | **Don’t know** | **Refused** |
| --- | --- | --- | --- | --- |
| a. High blood pressure | 1 £ | 2 £ | 99 £ | 88 £ |
| b. Heart disease | 1 £ | 2 £ | 99 £ | 88 £ |
| c. Diabetes | 1 £ | 2 £ | 99 £ | 88 £ |
| d. Cancer or any malignancy | 1 £ | 2 £ | 99 £ | 88 £ |
| e. Obesity | 1 £ | 2 £ | 99 £ | 88 £ |
| f. Asthma | 1 £ | 2 £ | 99 £ | 88 £ |
| g. Other, specify: gg. _______________ | 1 £ | 2 £ | 99 £ | 88 £ |

**If yes to any>>** Thank you, Some medications affect the body’s ability to regulate temperature. Could you tell me which medications (if any) you are currently taking?

**11.** Is there any chance that you are currently pregnant? [This is about physical thermoregulation, we are not asking about plans to have a baby.]

1. Yes
2. No
3. Don’t know
4. Refused

#### Sleep

Next, this section will ask about your sleep patterns, again, because sleep is often disrupted by heat

**12.** During the past month, how would you rate your sleep quality overall:

1. excellent

2. very good

3. good

4. fair

5. poor

99. Don’t know

88. Refused

**13**. The next questions ask about your sleep habits. Pick the answer that best describes how often you experienced the situation in the past 4 weeks.

|  | **No,**  **not in the last 4 weeks** | **Yes,**  **less than once a week** | **Yes,**  **1 or 2 times a week** | **Yes,**  **3 or more times a week** | **Don’t Know** | **Refused** |
| --- | --- | --- | --- | --- | --- | --- |
| a. Did you have trouble falling asleep? | 1 £ | 2 £ | 3 £ | 5 £ | 99 £ | 88 £ |
| b. Did you wake up two (2) or more times a night? | 1 £ | 2 £ | 3 £ | 5 £ | 99 £ | 88 £ |
| c. Did you feel overly sleepy during the day? | 1 £ | 2 £ | 3 £ | 5 £ | 99 £ | 88 £ |

**If yes to one or more items above>>**

Are there specific things in your environment that disrupt your sleep (e.g., noise, temperature, neighbors)?

#### Hydration

**14.** On a typical day, how many 8oz glasses of liquid (show picture of 8oz glass) do you consume:

[write in number for each, 0 if nothing]

- Coffee / Tea _____
- Milk ______
- Water _____
- Juice _____
- Alcohol ______
- Soda _____
- Other _____ (please specify)
- Refused

**15.** In general, do you feel that you drink enough water [circle response: Yes / no / Refused ]?

If no, why do you not drink enough water?

#### Adaptation

**16.** Generally speaking, what are the first three things (rank 1 to 3, 1 being the first) you do to cool down when your residence gets too hot?

___ Take off/Remove layers of clothing

___ Close/open blinds or shades

___ Open windows

___ Turn on air conditioner

___ Turn on portable fan

___ Turn on ceiling fan

___ Turn down thermostat

___ Leave the house for a cooler area

___ Other, specify ( _______________ )

88. Refuse to answer

**17.** Do you ever leave your house for a cooler area when your residence gets too hot? [ yes / no ]

**If yes,**

where do you usually go? Do you… [circle all that apply.]

1. Go to someone else’s air conditioned home.
2. Go to an air conditioned community center, library, or other public place.
3. Go to an air conditioned business.
4. Go to a park or area with tree shade
5. Other, specify ____________________
6. Don’t know
7. Refused

**If no,**

What is the most important reason you don’t leave home to find a cooler place during hot weather?

#### Heat wave education

**18.** How concerned are you that, on very hot days, the heat indoors could cause you to become ill?

1. Not at all concerned
2. Slightly concerned
3. Somewhat concerned
4. Moderately concerned
5. Extremely concerned
6. Refused

**19.** Can you tell me any symptoms you might expect from too much heat?

**20**. Do you worry about anyone else in your household, family, or community getting sick because of heat?

1. Yes
2. No
3. 88. Refuse to answer

**If yes,** can you tell us what makes you worried?

**21.** Last summer, or this summer, did you see, hear or read about any warnings about dangerously hot weather in Chelsea or Boston?

1. Yes
2. No
3. Don’t know
4. Refused

if Yes:

What was the source of this information __________________

**22.** What, if anything, do you typically do (or would you do) when you read or hear heat warning information?

#### Heat Illness

**23.** Did you or anyone in your household experience any of the following symptoms of heat illness during a particularly hot day last summer (or this summer)? [Read all and specify in table who in the household experienced symptoms]

| Household member  *Specify the relation to the participant* | a. Hot,dry skin OR cold, clammy skin | b. Confusion, hallucinations, disorientation | c. Light headedness, feeling faint or dizzy | d. Unconscious or unresponsive | e. Nausea or vomiting | f. Trouble breathing | g. Rapid, strong pulse | h. Muscle cramps | i. Weakness | j. Light headedness, feeling faint or dizzy | k. Headache | l. Rash |
| --- | --- | --- | --- | --- | --- | --- | --- | --- | --- | --- | --- | --- |
| 1. You (the person being interviewed) |  |  |  |  |  |  |  |  |  |  |  |  |
| 2. Relation to participant: _______________ |  |  |  |  |  |  |  |  |  |  |  |  |
| 3. Relation to participant: _______________ |  |  |  |  |  |  |  |  |  |  |  |  |
| 4. Relation to participant: _______________ |  |  |  |  |  |  |  |  |  |  |  |  |
| 5. Relation to participant: _______________ |  |  |  |  |  |  |  |  |  |  |  |  |
| 6. Relation to participant: _______________ |  |  |  |  |  |  |  |  |  |  |  |  |
| 7. Relation to participant:  _______________ |  |  |  |  |  |  |  |  |  |  |  |  |

FOR EACH PERSON WITH SYMPTOMS:

Where were you (or the other household member) when the symptoms occurred? [circle response]

- At home
- At another indoor location? Specify:
- Outside location? Specify:

Did you/this person visit a doctor or other medical professional for treatment of these symptoms?

( Yes / No / Don't Know / Refused)

If a medical professional was visited, was the diagnosis related to heat exposure?

If so, please describe the diagnosis (as it related to heat exposure).

### Transportation

**24.** What is your normal mode of transportation (to work, for groceries)?

**24a.** Has your normal transportation changed from last summer to this summer, due to the coronavirus?

If yes, elaborate:

**25.** Do your change your normal mode of transportation when it is hot?

1. Yes
2. No
3. Unsure
4. Refuse to answer

**If yes,** what changes do you make?

**If yes,** you make changes, how does these changes impact your commuting experience?

### Social Capital

**26.** What neighborhood do you live in?

**27.** Is your “neighborhood” different from your “community”? [Yes / no / Refused]

***If yes***, how so

**28.** Now I will read four statements about your neighborhood and the people who live there (as defined above) and you will choose the response that best represents your agreement. The options are strongly agree, agree, neither agree nor disagree, disagree, and strongly disagree. You may also select ‘no opinion.’

People are willing to help their neighbors.

1 ☐ Strongly agree 2 ☐ Agree 3 ☐ Neither agree nor disagree

4 ☐ Disagree 5 ☐ Strongly disagree 0 ☐ No opinion ☐ Refuse to answer

This is a close-knit neighborhood.

1 ☐ Strongly agree 2 ☐ Agree 3 ☐ Neither agree nor disagree

4 ☐ Disagree 5 ☐ Strongly disagree 0 ☐ No opinion ☐. Refuse to answer

People in this neighborhood can be trusted.

1 ☐ Strongly agree 2 ☐ Agree 3 ☐ Neither agree nor disagree

4 ☐ Disagree 5 ☐ Strongly disagree 0 ☐ No opinion ☐ Refuse to answer

People in this neighborhood don't get along with each other.

1 ☐ Strongly agree 2 ☐ Agree 3 ☐ Neither agree nor disagree

☐ Disagree 5 ☐ Strongly disagree 0 ☐ No opinion ☐ Refuse to answer

### Financial health questions

#### General

**29.** These next questions are about work. (Choose only one answer)

Are you currently?

1. £ Employed (for wages)

2. £ Self-employed

3. £ Out of work for 1 year or more **(skip to 31)**

4. £ Out of work for less than 1 year **(skip to 31)**

5. £ A Homemaker / Stay at home parent **(skip to 31)**

6. £ A Student **(skip to 31)**

7. £ Retired **(skip to 31)**

8. £ Unable to work **(skip to 31)**

88. £ Refused to answer **(skip to 31)**

99. □ Don’t Know

>> **if employed** then

On average, how many hours per week are you employed for pay?

1. □ < 20
2. □ 20 – 40
3. □ > 40

99. □ Don’t Know

88 £ Refused

**30.** Which of the following best describes your usual work schedule?

1. £ Day shift

2. £ Afternoon shift

3. £ Night shift

4. £ Split shift

5. £ Irregular shift/on call

6. £ Rotating shifts

99. £ Don’t Know

☐ Refuse to answer

**31**. Which category represents the total combined income of all members of this household during the past 12 months? This includes money from jobs, net income from business, rent, pensions, social security payments and any other money income received (by members of this household who are 15 years of age or older.)

1. □ Less than $ 24,999
2. □ $25,000 to $49,999
3. □ $50,000 to $74,999
4. □ $75,000 to $99,999
5. □ $100,000 or more

99. □ Don’t Know

88. □ Refused to answer

**32.** How many household members are supported by your total combined household income (including yourself)?

1. £ 1 6. □ 6

2. □ 2 7. □ 7-10

3. □ 3 8. □ 11 or more

4. □ 4 99. □ Don’t Know

5. □ 5 88 £ Refused

How many of those people are children?

(Please include anyone under 18 years or anyone older than 18 years & in high school)

(Enter No. of children) _____

99. □ Don’t Know

☐ Refuse to answer

#### Residential finances

**33.** In the last 12 months, on how many different occasions did you request maintenance or repairs to your residence?

1. £ 0
2. £ 1 - 4 requests
3. £ 5 – 7 requests
4. £ More than 8 requests

99. □ Don’t Know

☐ Refuse to answer

**34.** Do you or someone in your household pay for cooling and heating at this residence, separate from your monthly rent or mortgage?

1. □ Yes
2. £ No

99. □ Don’t Know

☐ Refuse to answer

#### Material hardship

**35.** At the present time, how easy or difficult is it to pay your bills?

1. Very difficult
2. Difficult
3. Neutral
4. Easy
5. Very easy
6. ☐ Refuse to answer

**36.** Do you sometimes have to make choices about which bills to pay during a given month?

1. Yes
2. No
3. Unsure
4. Refuse to answer

**37**. Do extreme temperatures impact your decision about which bills to pay?

- - 1. Yes
    2. No
    3. Unsure
    4. Refuse to answer

**If yes,** If you have to make choices about which bills to pay, how do you prioritize?

### Demographics

We are almost done. This is the final section of the questionnaire. We would like to ask you some demographic information.

**38.** What year were you born? __________ (YYYY)

88. £ Refused to answer

**39.** How do you identify your Gender? 1. £ Woman 2. £ Man 3. Gender Non-Binary 4. £ Other, specify: 3a. __________

99. □ Refused

**40.** What is the highest degree or level of school you have completed (in US or elsewhere)?

| A | Less than high school diploma or GED | 1 £ |
| --- | --- | --- |
| B | High school diploma or GED | 2 £ |
| C | Some college but no degree | 3 £ |
| D | Associate degree | 4 £ |
| E | Bachelor’s degree (i.e. BA, BS) | 5 £ |
| F | Post graduate degree (i.e. masters or doctoral) | 6 £ |
| G | Refused to answer | 88 £ |
| H | Don’t know | 99 £ |

**41.** Are you Hispanic or Latino/a?

| a | 1 £ Yes - Hispanic or Latino | A person of Cuban, Dominican, Mexican, Puerto Rican, Central American (ex. Belize, Guatemala, Honduras, El Salvador, Nicaragua, Costa Rica, Panama), South American (ex. Argentina, Brazil, Chile, Columbia, Ecuador, Peru, Venezuela), or other Spanish culture or Origin, regardless of race.  **If participants specifies from where, write in here:** __________ |
| --- | --- | --- |
| b | 2 £ No, Not Hispanic or Latino |  |
| c | 3 £ Other | Specify: cc. ____________________________ |
| d | 99. □ Don’t Know |  |
| e | ☐ Refuse to answer |  |

**42.** What is your race? (*Select all that apply):*

| a | American Indian or Alaska Native | 1 £ | A person having origins in any of the original peoples of North, Central, or South America, and who maintains tribal affiliations or community attachment (ex. Cherokee, Chippewa, Navajo, Sioux Tribal Groups.) |
| --- | --- | --- | --- |
| b | Asian | 2 £ | A person having origins in any of the original peoples of the Far East, Southeast Asia, or the Indian subcontinent including, Cambodia, China, Hmong, India, Japan, Korea, Laotian, Malaysia, Pakistan, the Philippine Islands, Thailand, and Vietnam. |
| c | Black or African American | 3 £ | A person having origins in any of the black racial groups of Africa. (Including: Haitian, African Caribbean, Cape Verdean, African ancestry, or other.) |
| d | Native Hawaiian or Other Pacific Islander | 4 £ | A person having origins in any of the original peoples of Hawaii, Guam, Samoa, or other Pacific Islands. (ex. Chamorro, Fijian, or Tongan) |
| e | White | 5 £ | A person having origins in any of the original peoples of Europe, the Middle East, or North Africa. |
| f | Unknown/not reported | 6 £ |  |
| g | Other | 7 £ | Specify: gg. __________________________________ |
| h | Don’t Know | 99. □ |  |
| i | ☐ Refuse to answer |  |  |

**43.** Were you born in the U.S.?

1. £ Yes
2. £ No
3. □ Refused to answer
4. □ Don’t know

**If No,** (born outside in US), how long have you lived in the U.S.?

1. £ >10 years
2. £ 5-10 years
3. £ 3-5 years, not including 5
4. £ 1-3 years, not including 3
5. £ Less than a year

99. £ Don’t know

☐ Refuse to answer

**44.** Where did you live before Chelsea/East Boston, if anywhere, and how long ago?

1. £ United States, specify: 1a. _______________

1. £ Elsewhere in North America, specify: 1a. _______________

2. £ Central America, specify: 2a. _______________

3. £ South America, specify: 3a. _______________

4. £ Asia, specify: 4a. _______________

5. £ Indian subcontinent, specify: 5a. _______________

6. £ Europe, specify: 6a. _______________

7. £ Africa, specify: 7a. _______________

8. £ Caribbean, specify: 8a. _______________

9. £ Other, specify: 9a. _______________

99. □ Don’t know

☐ Refuse to answer

## Weekly interview

**This brief questionnaire would be asked once per week during the study period, via phone call.**

1. Have you moved since we last checked in with you?

- No
- Yes

If yes… What is your new address?

(Outside of Chelsea/East Boston participant should withdraw.)

1. How hot or cold has your home felt over the LAST WEEK?

- Very Cold
- Cold
- Neutral/Comfortable
- Hot
- Very Hot
- N/A, have not been at home the last week

1. In terms of your own comfort level, over the LAST WEEK have you found your home to be:

- Much Too Cold
- Too Cold
- Comfortable
- Too Hot
- Much Too Hot
- N/A, have not been at home the last week

1. How dry or humid has your home felt over the LAST WEEK?

- Very Dry
- Dry
- Neutral
- Humid
- Very Humid
- N/A, have not been at home the last week

1. In terms of your own comfort level, over the LAST WEEK have you found your home to be:

- Much Too Dry
- Too Dry
- Comfortable
- Too Humid
- Much Too Humid
- N/A, have not been at home the last week

1. How would you characterize the quality of your sleep over the LAST WEEK?

- More disrupted than usual
- Same as usual
- Better than usual

1. This week, what were the first three things you did to cool down when your residence got too hot? *Rank from 1 first to 3 last.*

___ Take off Remove layers of clothing

___ Close/open blinds or shades

___ Open windows

___ Turn on air conditioner

___ Turn on portable fan

___ Turn on ceiling fan

___ Turn down thermostat

___ Leave the house for a cooler area

___ Other, specify ( _ ___ )

If checked “leave the house for cooler area” **Where did you go?**

1. Someone else’s air conditioned home.
2. An air conditioned community center, library, or other public place.
3. An air conditioned business.
4. Other, specify ____________________
5. Don’t know
6. Refused

If did not check “leave the house for a cooler area” **Under normal circumstances (not during a pandemic), would you leave the house if it were too hot?**

- No. **What are some reasons you would not leave home?**
- Yes. **Where would you go. for example?**

[Interviewer instructions: We are interested in their reasons for staying or leaving which could include, Don’t have transportation, Don’t feel safe leaving home, Are too sick to leave home, Would have to spend money leaving your home, Would rather not spend time with people you don’t know well, There are no activities at the air conditioned facilities, Don’t think the heat is really dangerous to you. Prefer to stay home…]

8. Did you use air conditioning in your home over the last week?

- No.
- Yes.

IF YES: Over the last week, on how many days did you run an air conditioner (or central AC system) in your home? 🡪

On the days when an air conditioner or central AC system was used in your home, for how many hours per day was it turned on (on average)?

a. Number of hours in the morning (8AM-12PM) __________ [min=0; max=4] □ Don’t Know

b. Number of hours in the afternoon (12 PM- 4 PM) _________ [min=0; max=4] □ Don’t Know

c. Number of hours in the evening (4 PM-10PM) _________ [min=0; max=6] □ Don’t Know

d. Number of hours at night (10 PM-8AM) __________ [min=0; max=10] □ Don’t Know

9. Recall the hottest day in the last week. On this day, how often did your air conditioner (or central AC system) cool your home to a level that was comfortable for everyone in the household?

- all of the time/day
- most of the time/day
- some of the time/day
- hardly ever
- never
- Unsure

10. 🡪 Why? 🡪

**Extreme Heat Illness and Heat Stress Symptoms**

11. Did anyone in your household (including yourself) experience any of the following symptoms of heat illness over the last week?

- Hot, dry skin OR cold, clammy skin
- Confusion, hallucinations, disorientation
- Unconscious or unresponsive
- Nausea or vomiting
- Trouble breathing
- Rapid, strong pulse
- Muscle cramps
- Weakness
- Light headedness, feeling faint or dizzy
- Headache
- Rash

| Household member  *Specify the relation to the participant* | a. Hot,dry skin OR cold, clammy skin | b. Confusion, hallucinations, disorientation | c. Light headedness, feeling faint or dizzy | d. Unconscious or unresponsive | e. Nausea or vomiting | f. Trouble breathing | g. Rapid, strong pulse | h. Muscle cramps | i. Weakness | j. Light headedness, feeling faint or dizzy | k. Headache | l. Rash |
| --- | --- | --- | --- | --- | --- | --- | --- | --- | --- | --- | --- | --- |
| 1. You (the person being interviewed) |  |  |  |  |  |  |  |  |  |  |  |  |
| 2. Relation to participant: _______________ |  |  |  |  |  |  |  |  |  |  |  |  |
| 3. Relation to participant: _______________ |  |  |  |  |  |  |  |  |  |  |  |  |
| 4. Relation to participant: _______________ |  |  |  |  |  |  |  |  |  |  |  |  |
| 5. Relation to participant: _______________ |  |  |  |  |  |  |  |  |  |  |  |  |
| 6. Relation to participant: _______________ |  |  |  |  |  |  |  |  |  |  |  |  |
| 7. Relation to participant:  _______________ |  |  |  |  |  |  |  |  |  |  |  |  |

12. Where were you / the affected person when the symptoms occurred?

- At home
- At another indoor location (specify below)
- Outside location (specify below)

13. Did you / the affected person visit a doctor or other medical professional for treatment of these symptoms? (Yes/ No/Don't Know)

## Exit Interview

Thank you for participating in the full study this summer! Thanks for taking the time to join us to talk about your experience participating in this study, as well as your experiences coping with heat over the summer.

You've probably noticed that I am recording the session. This is because I don't want to miss any of your comments.

*Overall*

- How has been your experience of participating in this study been overall?

We asked you to do a lot: carry and wear and put up in your home 4 different devices, sync data continuously, check in every week. We want to document your experiences so we can learn from you and improve for the next time we do this work. We appreciate your candid feedback!

- What were the main challenges or barriers of participating?
- *Potential Challenges:*
  - *issues with internet access, wifi? what would it take to improve that?*
  - *technology proficiency: unfamiliar with using phone apps? phone apps with hard to use interfaces? how to use a phone itself. (knowing how to “swipe through”, even the “intuitive” features of touchscreen use - might not have been)*
    - *Sometimes apps are user friendly and sometimes it's difficult to navigate. How did you find your experiences using these apps? How comfortable/familiar or uncomfortable/new were these experiences ...*
  - *phone issues? (data, memory, storage.?)*
  - *navigating the apps (uploading data)*
    - *did the syncing become easier over time?*
  - *the weekly calls*
    - *were they burdensome? hard to schedule? difficult to remember? hard to find a quiet place to take the call?*
    - *→ did we mainly use video or no video? why? (slow connection?)*
- *would it have been easier in person?*
  - *Was there any particular challenge or burden for you with the Fitbit, HOBO, or Tile or any app in particular?*
  - *issues with functioning devices - HOBOs stopping, FitBits uncomfortable.*

Now I will ask about how you used the devices we provided for this study.

- Did you carry the HOBO/Tile with you every time you went out? Options: Always, Most of the time, Some of the time, Never
- Did you wear the FitBit: Every night, most nights, some nights, never?

Had you used Zoom before this study? Did you mainly use a computer or phone to zoom/check in?

- What about the main facilitators to participating – the things that made it easy to participate?
  - *The calls, were they helpful?*
    - *with syncing devices/ apps*
    - *anything else?*
  - *Zooming to sync/upload data?*
    - *Zoom vs phone call - video vs audio*

*Coping with summer heat*

- What were the biggest challenges to coping with heat this summer?
- What were the biggest challenges to maintaining a comfortable home environment this summer?
  - *utilities cost?*
  - *AC old*
  - *when you used your home cooling strategies (AC, fan, taking off layers) and they weren’t enough, why not? what more would have been helpful to cooling your home?*
- What were the biggest challenges to experiencing comfortable temperatures when you were NOT at home this summer?
  - *at work? in transit?*
- How were your experiences different this summer, if they were different, compared to other summers in the past?

*Learning/Reflections*

- What, if anything, did you learn from this experience?
- What other reflections on your own experience of heat this summer would you like to share with us?
- Do you have any other reflections on this process that we didn’t ask about which you would you could share with us? We are hoping to do this study again, with modifications, and value your feedback.
  - What are your recommendations for the research team?
- Once we have analyzed all of this data that you have helped us collect, we will report back results to you. We also plan to summarize the results across all participants and share what we learn with city officials and decision-makers.
  - If the city had unlimited funds and resources for addressing the impacts of heat, what would you like to see in your community?
    - What would you like the city to do to help you cope with the burden of heat in your daily life?

*Misc…*

- Do you have any concerns about the cold/ heating season?
- How many floors are in your home/ on which floor do you live?
- *Smoking questions:*

Do you now smoke cigarettes every day, some days or not at all?

Every day

Some days

Not at all

Refused

Does anyone who lives in your residence smoke inside?

Yes

No, not at all **(skip next**)

Don’t Know **(skip next**)

How many people live here and smoke inside this residence?

Number of people smoking inside the residence ______________

Don’t Know

Do visitors to your residence ever smoke in your residence?

Yes

No

Do you or anyone in your household smoke e-cigarettes?

Yes

No

Don’t Know

- As you know, GreenRoots is one of the organizations leading this study. Could we give your email address to GreenRoots so they can include you on the email list?
- If we create a periodic email update or newsletter about the study, would you like to receive that? If so, email address..
- We may do a follow-up study, similar to this one, next summer. We will also be doing a Photovoice study next summer, which will involve taking photos of your experiences and discussing them with a small group, then publicly displaying those photos. Are you interested in participating in potentially participating in one or both of these studies?
  - *response options: photovoice, repeating this study, or both*

As a final question, I will review the TILE data we have collected with you. I want to ask if what we have collected is accurate to your experiences, and if there are some days or times that look inaccurate.

1. Merry and Bettinger, “Smartphone GPS Accuracy Study in an Urban Environment.” [↑](#footnote-ref-1)
2. Bent et al., “Investigating Sources of Inaccuracy in Wearable Optical Heart Rate Sensors.” [↑](#footnote-ref-2)
3. Tedesco et al., “Validity Evaluation of the Fitbit Charge2 and the Garmin Vivosmart HR+ in Free-Living Environments in an Older Adult Cohort.” [↑](#footnote-ref-3)
4. Haghayegh et al., “Accuracy of Wristband Fitbit Models in Assessing Sleep: Systematic Review and Meta-Analysis.”

   Note: These citations all appear in the main text [↑](#footnote-ref-4)
